# The Perils of Straying from Protocol: Sampling Bias and Interviewer Effects

**DOI:** 10.1371/journal.pone.0118025

**Published:** 2015-02-18

**Authors:** Carrie J. Ngongo, Kevin D. Frick, Allen W. Hightower, Florence Alice Mathingau, Heather Burke, Robert F. Breiman

**Affiliations:** 1 Centers for Disease Control and Prevention/Kenya, Nairobi, Kenya; 2 The Johns Hopkins Carey Business School, Baltimore, Maryland, United States of America; 3 Centers for Disease Control and Prevention, Nairobi, Kenya; 4 Kenyatta University School of Public Health, Nairobi, Kenya; David Geffen School of Medicine at UCLA, UNITED STATES

## Abstract

Fidelity to research protocol is critical. In a contingent valuation study in an informal urban settlement in Nairobi, Kenya, participants responded differently to the three trained interviewers. Interviewer effects were present during the survey pilot, then magnified at the start of the main survey after a seemingly slight adaptation of the survey sampling protocol allowed interviewers to speak with the “closest neighbor” in the event that no one was home at a selected household. This slight degree of interviewer choice led to inferred sampling bias. Multinomial logistic regression and post-estimation tests revealed that the three interviewers’ samples differed significantly from one another according to six demographic characteristics. The two female interviewers were 2.8 and 7.7 times less likely to talk with respondents of low socio-economic status than the male interviewer. Systematic error renders it impossible to determine which of the survey responses might be “correct.” This experience demonstrates why researchers must take care to strictly follow sampling protocols, consistently train interviewers, and monitor responses by interview to ensure similarity between interviewers’ groups and produce unbiased estimates of the parameters of interest.

## Introduction

Survey data allow public health professionals to draw conclusions about the health realities of entire populations. Sampling is critical because the underlying truth is often impossible to measure directly. Yet errors arise when the observed value of a phenomenon differs from the true value of that phenomenon [1].

Bias describes errors that would be expected to occur on every administration of the same survey design, while variable errors would be expected to vary across survey administrations [1]. For example, sampling bias prevails when some members of the intended study population are less likely to be included than others.

One source of possible measurement error relates to the interviewers who administer household surveys. Interviewers can affect respondents’ answers in several ways. The presence of an interviewer can prompt respondents to take social norms into account when answering questions [2]. Respondents may “edit” their answers before communicating [3] based on their understanding of prevailing social norms and how they imagine the interviewer will perceive them. Further, respondents may be influenced by the interviewer’s observable characteristics, such as age, ethnicity, and gender, as they formulate answers or judge which answers would be socially appropriate [2]. Such interviewer-related measurement error is role-independent. Error can also be role-dependent, based on how interviewers ask survey questions [1]. The interviewer’s verbal and nonverbal presentation can affect respondents’ answers and perceptions of approval/disapproval [2].

To minimize the risk of interviewer measurement error, surveys would ideally include large numbers of interviewers and respondents, with an interpenetrated survey design that randomly assigns interviewers to respondents. Yet this is not always practical, particularly when investigators set out to study an outcome unrelated to interviewer error.

Contingent valuation has been embraced as a tool to anticipate the demand side of market behavior in situations where markets to satisfy potential demand do not yet exist. “Willingness to pay” surveys (WTP) require respondents to consider how they would behave in a theoretical market and place monetary value on a diverse assortment of health care goods and services [4]. Although this evaluation tool has been applied in diverse settings, questions persist about the validity and reliability of willingness to pay surveys [5, 6, 7]. A variety of study design issues can contribute to bias in the data that are gathered using these methods: the construction and specification of the hypothetical market, the administration of the survey, and the analysis and interpretation of the data [8].

Much has been written about how to mitigate the threat of bias in contingent valuation surveys. The scenario must be as realistic as possible, with comprehensible descriptions of the risks and treatment [6, 8]. The questioning format for eliciting values should minimize leading respondents in any way [8, 9]. Care should be taken that there are no perceived incentives for respondents to misrepresent their true WTP amounts [10]. Interviewers should be independent and trained to present the willingness to pay data elicitation questions identically and consistently. Once the data have been gathered, the analytic process should include tests for potential bias [8]. Even when contingent valuation surveys have been designed and executed with great care, it is prudent to treat hypothetical statements about behavior with caution when drawing policy conclusions [6].

After striving to minimize possible sources of bias in their contingent valuation studies, investigators are encouraged to evaluate the validity of their findings and to consider the relationship between the findings and underlying hypotheses. Does what was measured coincide with what was supposed to be measured? Are the results similar to past valuation of the same and similar goods? Do the measurements match what was expected or predicted? Could any lingering bias render the findings invalid?

## Methods

This investigation took place in the informal urban settlement of Kibera in Nairobi, Kenya. Kibera is characterized by poverty, suboptimal sanitation and water provision, structures built of mud with corrugated metal roofs, and high incidence of infectious diseases [11, 12]. In order to learn more about population health in Kibera, the Centers for Disease Control and Prevention (CDC) in Kenya and the Kenya Medical Research Institute (KEMRI) have collaborated to conduct active, population-based infectious disease surveillance (PBIDS) since 2005 [13]. Approximately 28,000 Kibera residents who participate in the surveillance program receive free primary health care services at the Tabitha Clinic, which is operated by the non-governmental organization Carolina for Kibera, Inc. in partnership with the CDC and KEMRI. The Tabitha Clinic hoped to use information about the value its clients place on basic health services to inform a pricing structure for its non-subsidized patients. Contingent valuation data were also sought for novel vaccines that may be introduced in Kibera in the future.

In preparation for the willingness to pay survey, focus group discussions were conducted to ascertain qualitative background about the community’s perceptions of disease severity and risk as well as to determine which assets should be included in a locally appropriate proxy for household wealth [14]. Using information from the focus group discussions, a survey was designed to elicit willingness to pay information about seven health care goods or services of interest to the Tabitha Clinic: general consultation (i.e. visit to a clinician), malaria testing, chest x-ray, a product for chlorinating drinking water (Waterguard), and vaccines against diarrhea, pneumonia, and typhoid.

One male and two female bilingual (Kiswahili/English) interviewers from Kibera were recruited and trained on the contingent valuation rationale and the use of the survey instrument. During training, each interviewer practiced conducting the survey with trainers and with one another to receive feedback and improve consistency. A three-week-long field pilot with more than 200 interviews provided further practice as well as the opportunity to hone the survey instrument. Throughout the survey period a bilingual field manager routinely accompanied each interviewer into respondents’ homes to supervise the conduct of interviews. One female interviewer resigned before the end of the data collection phase, which meant she conducted fewer interviews than the others.

The PBIDS program enumerated households in its catchment area sequentially, such that household numbers corresponded to location. Every ninth household identification number was selected from the database household list, ensuring a geographically representative sub-sample. The selected household numbers were assigned randomly to the interviewers. During the field pilot, interviewers were instructed to visit each household on their list, proceeding to the next household in the event that they found no one at home. In practice, however, this meant that interviewers spent more time walking from one house to the next than actually conducting interviews. In response, the investigators agreed early in the main survey’s implementation that interviewers could talk with either a member of the selected household or with the closest neighbor they found. In this way, the list of households was treated as a series of intentionally sampled geographic points.

Enrolled participants in PBIDS who were >18 years old were eligible to respond to the survey; this eligibility was understood by all interviewers. Once identified, eligible subjects were asked if they would like to respond to the questionnaire, and informed written consent was obtained. The protocol and consent forms were reviewed and approved by the KEMRI ethical review board (SSC 1148). Data were collected between November 2006 and March 2007.

To proxy for household wealth, interviewers asked or observed whether respondents owned a phone, radio, television, bicycle, bed, seat other than a stool, and furniture (defined as a large cupboard, wall unit, or wardrobe). Interviewers also observed housing characteristics, including reinforced walls (as opposed to mud or iron sheets alone), cement flooring (as opposed to mud), the presence of a ceiling (either homemade or landlord-installed), existence of electricity, access to a shared latrine in the compound, the use of kerosene, electricity, or gas for cooking (as opposed to charcoal, recycled charcoal, or wood), and whether or not respondents were landlords. Principal components analysis was carried out using these assets. Households were assigned to the bottom third or the top two-thirds of the socio-economic spectrum for purposes of using a dichotomous variable for analysis. Demographic information regarding each participant’s age, literacy, and education level was self-reported.

Contingent valuation data were analyzed using Stata software (StataCorp, 2007). The planned analysis was not possible, however, because the strongest predictor of WTP response was interviewer. We therefore performed multinomial logistic regression on both the main survey and the field pilot data to compare respondents who talked with one of the two female interviewers (F1 and F2) with respondents who talked with the one male interviewer (M1). Post estimation commands compared F1’s respondents with F2’s.

The regressions considered all of the demographic variables assessed, including respondents’ socio-economic status, sex, and education level, the sex of the household head, whether the respondent had visited the Tabitha Clinic in the previous six months, whether there was a child under five years in the household, whether the respondent could borrow money if needed, and the number of people the respondent would consult before making a decision. We tested whether the relative risk ratios were different from one and different from each another for the statistically significant variables: socio-economic status, sex, whether the respondent had visited the Tabitha Clinic in the previous six months, whether the household had children under five years old, whether the respondent could borrow money if needed, and the number of people the respondent would consult before making a decision.

Regressions on the outcomes of interest tested the consistency of the interviewer effects. Dummy variables were created to account for interviewer effects in these models. Models included individual terms for interviewers (confounding) or interaction terms that allowed the interviewer effect to depend on the value of the explanatory variable.

## Results

The 805 main survey participants tended to be young (mean age = 25, SD = 7.50) and mostly female (84.3%). Nearly all were literate (95.5%); most lived with children under five years old (79.2%) (Table 1). Education level ranged from none to higher education; the modal education level completed was standard eight (eighth grade, 54.1%). Most respondents reported that they could borrow money in case of need (75.9%). Of the 79.7% of respondents that reported having access to a latrine, the mean number of neighbors sharing the latrine was 35.6 and the median was 30; 20.3% of respondents reported that they do not have any access to a latrine. Latrine access is an important indicator of socio-economic status in contexts featuring notoriously poor sanitation such as Kibera.

**Table 1:** Sample demographic characteristics.

	% (n), N = 805
Median age (SD)	25 (7.50)
Male	15.6 (126)
Female	84.3 (679)
Literacy	97.5 (781)
Highest education level achieved:	
Never attended school	1.6 (13)
Some primary school	29.9 (240)
Completed standard eight (eighth grade)	54.1 (435)
Completed form four (high school)	13.3 (107)
College/post-secondary	1.1 (9)
Male-headed household	89.2 (713)
Female-headed household	10.8 (86)
Median number in household (SD)	5 (2.37)
Households with children under five	79.2 (638)
Respondents who report they can borrow money in case of need	75.9 (610)

Table 2 describes asset ownership, a proxy for wealth that would be expected to be associated with willingness to pay. The majority of respondents had electricity (63.6%), radio (86.0%), television (52.2%), and mobile phone access (62.1%). The majority of homes had cement flooring (86.3%), and the modal type of wall was mud reinforced with cement (74.9%). Just 1.4% of respondents lived in households with landlord-installed ceilings, while 296 (36.8%) had a home-made ceiling and 498 (61.9%) had none.

**Table 2:** Sample asset ownership.

	% (n), N = 805
Electricity	63.6 (512)
Radio	86.0 (692)
Television	52.2 (420)
Color	31.8 (256)
Black and white	20.4 (164)
Mobile phone	62.1 (500)
Self	37.0 (298)
Spouse	25.1 (202)
Bicycle	7.3 (59)
Bed	98.0 (789)
Landlord	4.0 (32)
Cement flooring	86.3 (695)
Ceiling	38.1 (307)
Landlord-installed	1.4 (11)
Home-made	36.8 (296)
Latrine access	79.7 (641)
Mean number sharing latrine (SD)	35.6 (23.4)
Cooking fuel	
Kerosene, electricity, or gas	73.6 (592)
Charcoal	21.8 (175)
Recycled charcoal or wood	4.4 (35)
None	0.2 (2)
Seats	
Butterfly sofa set	5.6 (45)
Wooden sofa set with cushions	62.1 (500)
Wooden sofa set without cushions	8.6 (69)
Metal sofa set with cushions	10.3 (83)
Metal sofa set without cushions	2.0 (16)
Plastic chairs	1.0 (8)
Stool	9.8 (79)
None	0.6 (5)
Furniture	
Wall unit	6.0 (48)
Wardrobe	1.5 (12)
Large cupboard	26.9 (216)
Small cupboard	8.8 (71)
Clothes rack	1.4 (11)
None	55.5 (446)
Walls	
Cement	0.3 (2)
Wood	2.8 (22)
Mud reinforced with cement and painted	3.9 (31)
Mud reinforced with cement	74.9 (579)
Mud reinforced with iron sheets	1.5 (12)
Iron sheets	2.8 (22)
Mud	16.2 (129)

The multinomial logistic regression analysis on 145 field pilot records with M1’s sample as the base did not generate any statistically significant relative risk ratios (Table 3). Post-estimation tests revealed whether the two female interviewers encountered participants of the same socio-economic profile as M1 and whether the two female interviewers encountered participants of the same socio-economic profile as one another. The chi-squared value for the first test was 6.81, with an associated probability of 0.0331. The chi-squared value for the second test was 6.71, with an associated probability of 0.0096.

**Table 3:** Field Pilot: Multinomial Regression comparing female interviewers to M1.

F2	RRR	P	95% CI	
female	1.53	0.38	0.60	3.91
number before making a decision > 1	0.86	0.50	0.57	1.32
children under five	0.62	0.30	0.25	1.52
clinic attendance over previous six months	1.40	0.53	0.49	3.97
could borrow money in case of need	1.09	0.87	0.40	2.93
high socio-economic status	2.63	0.057	0.97	7.09
F1				
female	2.11	0.16	0.75	5.93
number before making a decision > 1	0.89	0.58	0.60	1.33
children under five	1.39	0.52	0.51	3.75
clinic attendance over previous six months	0.46	0.12	0.17	1.22
could borrow money in case of need	0.45	0.09	0.18	1.13
high socio-economic status	0.66	0.35	0.28	1.58

In the multinomial logistic regression analysis on 779 main survey records with M1’s sample as the base, five of the relative risk ratios associated with demographic variables were statistically significant for interviewer F2 and six relative risk ratios associated with demographic variables were statistically significant for interviewer F1 (Table 4).

**Table 4:** Main survey: Multinomial Regression comparing female interviewers to M1.

F2	RRR	P	95% CI	
female	4.81	0	2.45	9.49
number before making a decision > 1	0.39	0	0.25	0.63
children under five	3.37	0.002	1.88	6.07
clinic attendance over previous six months	2.17	0.014	1.17	4.02
could borrow money in case of need	21.02	0	9.73	45.41
high socio-economic status	7.72	0	4.89	12.19
F1				
female	1.05	0.904	0.47	2.37
number before making a decision > 1	0.23	0	0.10	0.51
children under five	2.39	0.044	1.02	5.57
clinic attendance over previous six months	0.37	0.005	0.18	0.74
could borrow money in case of need	0.36	0.005	0.18	0.73
high socio-economic status	2.80	0.005	1.37	5.72

Compared with M1’s sample, F1’s respondents were less likely to consult two or more people before making a decision (RRR = 0.23, p = 0.000). Participants were one-third as likely to speak with F1 if they had visited the clinic in the past six months (RRR = 0.37, p = 0.005) or reported they could borrow money from neighbors in case of need (RRR = 0.36, p = 0.005). Those who were of high socio-economic status had a 2.8 times higher relative risk of speaking with F1 than with M1 (p = 0.005). Those with children under five at home had a 2.4-times higher relative risk of speaking with F1 than with M1 (p = 0.044).

Compared with M1’s sample, participants who spoke with F2 were 4.8 times more likely to be female (p = 0.000), 3.4-fold more likely to have children under five at home (p = 0.002), 2.2-fold more likely to have been to the clinic in the past six months (p = 0.014), and 7.7-fold more likely not to have been in the bottom third of the socio-economic spectrum (p = 0.000). F2’s respondents were less likely to consult two or more people before making a decision (RRR = 0.39, p = 0.000) and far more likely to report being able to borrow money in case of need (RRR = 21.0, p = 0.000).

Post-estimation tests revealed whether the two female interviewers encountered participants of the same socio-economic profile as M1 and whether the two female interviewers encountered participants of the same socio-economic profile as one another. The chi-squared value for the first test was 81.42, with an associated probability of 0.0000. The chi-squared value for the second test was 5.95, with an associated probability of 0.0147.

Tests of willingness to pay including dichotomous F1 and F2 interviewer variables demonstrated that F1 and F2 had similar estimates regarding general consultation, both significantly higher than M1 (Table 5). Results varied by willingness-to-pay outcome. The inclusion of interviewer-SES interaction terms did not result in statistically significant coefficients.

**Table 5:** Willingness to pay for general consultation (n = 306), with M1 as the reference.

	Coefficient	P	95% confidence interval	
high socio-economic status	39.85	0.143	13.57	93.27
interviewer F2	179.63	0.000	128.07	231.18
interviewer F1	200.12	0.001	86.19	314.05
Constant	30.73	0.135	9.6	71.06

*interviewer/SES interaction terms were not statistically significant when included in the model

** test whether F1 = F2: F = 0.12 and Prob > F = 0.7278

## Discussion

Existing sources differ widely in their description of Kibera’s demographic and socio-economic characteristics. Journalistic and internet sources at the time of this study estimated the density of the Kibera population as 826 people per hectare [15], 1,200 people per hectare [16], and 3,000 people per hectare [17]. KEMRI-CDC has determined population-density within the PBIDS area to be 770 per hectare [13]. The variation in reported numbers may lead researchers and members of the public to draw wildly different conclusions about Kibera’s demographics according to their information source. Our findings on the demographic characteristics and asset ownership of residents may complement recent census determinations to improve descriptions of the Kibera population.

It was not possible to draw any conclusions regarding participants’ willingness to pay for the health services of interest in this study due to the deep differences between interviewer samples. Each of the three interviewers unwittingly spoke with a different cross-section of the Kibera population. “Interviewer” thus became the biggest predictor of willingness-to-pay outcome and we abandoned the effort to glean reliable WTP-related data from the survey.

Interviewer M1 spoke with poorer respondents than either FI or F2, the female interviewers. The two female interviewers both were more likely to speak with respondents with children under five at home. Beyond this, the samples of the two female interviewers were more divergent than similar: Most respondents speaking with F2 reported being able to borrow money in case of need, while respondents did not similarly assert an ability to borrow when speaking with F1. F2 met with respondents who had visited the clinic in the past six months more than twice as often as M1, who in turn met with clinic-goers nearly three times more often than F1.

Inadequate training, faulty execution, and poor supervisory oversight can each lead to the collection of unreliable data resulting in non-random differences. Yet the three interviewers benefitted from field pilot experience and received ongoing supervision and mentorship while the survey was underway. They understood the importance of consistency in survey presentation. All three interviewers were Kibera residents, selected in the hope that their status as a neighbor would make them acceptable to survey respondents.

Sampling bias could explain the non-random differences. Our sampling approach was initially designed to ensure that interviewers would all meet similar populations, but it was adapted early in the main survey’s execution to reduce the time interviewers spent trudging from one empty home to another. Interviewers were instructed to talk with the closest neighbor they found if no one was home at the selected household, without any anticipation that there would be multiple options for “closest neighbor.” The slight level of interviewer choice fundamentally altered our sampling.

Presumably some respondents were from households that were sampled as the protocol directed, while others were “closest neighbors.” Complete information about which respondents were properly or improperly sampled during the main survey is unavailable. The field pilot faithfully adhered to the original sampling protocol, however, so a comparison of the field pilot and main survey data can be instructive.

One would expect the larger sample size of the main survey to deflate the standard errors while not changing the relative risk ratios of the pilot data. While the general trends are similar between the pilot and the main survey, the relative risk ratios are more pronounced in the main survey analysis. Compared with M1’s sample, participants who spoke with F2 were 1.5 times more likely to be female during the pilot but 4.8 times more likely to be female during the main survey. They were 2.6 times more likely to be of high socio-economic status during the pilot but 7.7 times more likely to be of high socio-economic status during the main survey. Again compared with M1’s sample, participants who spoke with F1 were 1.4 times more likely to have children under five during the pilot but 2.4 times more likely to have children under five during the main survey. While these data to not demonstrate the effect of improperly sampled households during the main survey, they indicate that the revised sampling process did drive much of the observed effect of interviewers on respondents.

Why were F2’s respondents 2.6 times more likely to be of high socio-economic status than those of M1 during the field pilot? The revised sampling approach cannot explain this. While interviewers may have had a choice of who to interview when they entered an enumerated household with multiple adults at home, this would not be expected to affect an SES measure based on durable assets. Although the enumerated households were randomly assigned to the interviewers, they are gradations in relative SES status within Kibera and it is possible that interviewer F2 simply worked in a higher-SES area.

The situation is less clear for questions that required reflection, such as whether respondents could borrow money in case of need or whether they had attended the clinic in the past six months. Literacy, education level, and the number who would be consulted before making a decision were all self-reported, opening the possibility that people simply responded in different ways to the different interviewers.

Gender is one of the most identifiable interviewer characteristics, so one might suspect that gender played a role: Respondents answered the male differently than the females. Or, since many of the demographic variables were directly observable, in a given household the male interviewer would be drawn to male respondents and the female interviewers would be drawn to respondents with children under five at home, by nature of their gender. Yet stratification by interviewer did not reveal a clear gender-related pattern. The differences in F1’s and F2’s responses throw caution on interpreting the findings through a gender lens alone. In the pilot data, the post-estimation tests indicate that F1 and F2 were as dissimilar to one another as either female interviewer was to M1. Respondent differences were more pronounced between M1 and F2 than between M1 and F1.

“Interviewer effects” broadly refer to measurement error attributable to a specific interviewer characteristic. In many cases, however, it may not be possible to determine which interviewer characteristic is driving the observed effect. It is impossible to separate the role of gender from interviewers’ personalities or other traits. The literature is inconclusive about whether data collected by male or female interviewers is more accurate [18], or if and when it would be appropriate to match interviewers and respondents by gender [1].

Our data suggest two interviewer-related difficulties. First, individuals respond differently to different interviewers, and interviewer effects have the potential to distort results no matter how well a protocol is respected. Second, the adaptation of the study protocol that allowed interviewers to identify “closest neighbors” in the event that no one was home introduced a level of interviewer choice, leading to interviewer-driven sampling bias.

Frequent data review might have brought these issues to light during the data collection phase. Although we assessed whether the data were clean and complete and ran preliminary WTP analyses during the data collection phase, we did not notice the demographic differences between interviewers’ samples until it was too late. Early detection would have allowed us to revert to a high-fidelity version of the sampling process. Statistically “fixing” by forcing similar characteristics over the entire sample would not be appropriate.

Interviewer effects are real and often hard to avoid. Well-written questions and high quality supervision cannot entirely mitigate the inherent weaknesses of human communication. Investigators would do well to compare their responses by interviewer to test for interviewer effects. When the number of subjects per interviewer is large, including an indicator for the interviewer can demonstrate whether other measured and inferred relationships change.

Although these data were collected for a contingent valuation survey, the issue of an interviewer effect on respondents is not specific to a particular research topic or methodology. Imperfect implementation can ruin any survey. This analysis of differences between interviewer samples demonstrates the importance of fidelity to research protocols. We recognized from the outset that interviewers must be as identical as possible in their presentation of surveys: Deep differences between interviewers related to gender, familiarity, or personality can create nuanced, systematic bias. The risk is particularly acute for studies involving a small number of interviewers and a relatively small number of respondents. Our experience highlights the importance of ensuring that sampling protocols consider and mitigate the myriad possibilities for interviewers to encounter fundamentally different populations, thereby thwarting hopes of reliable survey data.
